# Food related processes in the insular cortex

**DOI:** 10.3389/fnhum.2013.00499

**Published:** 2013-08-23

**Authors:** Sabine Frank, Stephanie Kullmann, Ralf Veit

**Affiliations:** ^1^Institute of Medical Psychology and Behavioral Neurobiology, University of TübingenTübingen, Germany; ^2^fMEG Center, University of TübingenTübingen, Germany; ^3^Institute for Diabetes Research and Metabolic Diseases of the Helmholtz Center Munich at the University of TübingenTübingen, Germany; ^4^German Center for Diabetes ResearchNeuherberg, Germany

**Keywords:** insular cortex, food, gustatory, neurofeedback, obesity, weight loss, eating disorders

## Abstract

The insular cortex is a multimodal brain region with regional cytoarchitectonic differences indicating various functional specializations. As a multisensory neural node, the insular cortex integrates perception, emotion, interoceptive awareness, cognition, and gustation. Regarding the latter, predominantly the anterior part of the insular cortex is regarded as the primary taste cortex. In this review, we will specifically focus on the involvement of the insula in food processing and on multimodal integration of food-related items. Influencing factors of insular activation elicited by various foods range from calorie-content to the internal physiologic state, body mass index or eating behavior. Sensory perception of food-related stimuli including seeing, smelling, and tasting elicits increased activation in the anterior and mid-dorsal part of the insular cortex. Apart from the pure sensory gustatory processing, there is also a strong association with the rewarding/hedonic aspects of food items, which is reflected in higher insular activity and stronger connections to other reward-related areas. Interestingly, the processing of food items has been found to elicit different insular activation in lean compared to obese subjects and in patients suffering from an eating disorder (anorexia nervosa (AN), bulimia nervosa (BN)). The knowledge of functional differences in the insular cortex opens up the opportunity for possible noninvasive treatment approaches for obesity and eating disorders. To target brain functions directly, real-time functional magnetic resonance imaging neurofeedback offers a state-of-the-art tool to learn to control the anterior insular cortex activity voluntarily. First evidence indicates that obese adults have an enhanced ability to regulate the anterior insular cortex.

## The insular cortex—from neuroanatomy to function

The insular cortex is embedded in the lateral sulcus of the mammalian brain. On the basis of cytoarchitectonic studies using myelin staining techniques, the insula can be subdivided in three major compartments according to the laminar structure, referred to as the anterior ventral agranular, dorsal anterior dysgranular, and posterior granular part of the insular cortex (Mesulam and Mufson, 1985; Gallay et al., 2012). The agranular anterior insula in junction to the caudal orbitofrontal cortex (OFC) and the adjacent frontal operculum has been identified as the primary taste cortex (Rolls, 2006). Besides multiple perceptive inputs of gustational cues (smell, taste, temperature, viscosity, texture) in the anterior insula and hence different pathways, additional granular and dysgranular regions especially the dorsal mid-insula are involved in gustation (De Araujo and Simon, 2009; Kurth et al., 2010). Their close interconnections with the OFC indicate that this part plays a predominant role in the evaluation of motivational states and primary reinforcers (Wager and Barrett, 2004). Also functional connectivity based analyses highlight the anterior part of the insular cortex as a major hub in cerebral processing of cognitive, emotional, motivational, and sensory stimuli, and, defines together with the anterior cingulate cortex (ACC) the salience network (Menon and Uddin, 2010). The anterior dysgranular part is superior to the agranular part bounded on the border to the frontal operculum. This part is particularly engaged during tasks requiring executive control, shifting attention, and working memory (Wager and Barrett, 2004). The intermediate part of the insula and its dysgranular laminar structure extending into the parietal operculum is strongly connected with all parts of the insula and is involved in motor, somatosensory, and pain processing (Kurth et al., 2010). Hence, neuroanatomical findings indicate that the insular cortex is an important structure on the transition between allocortex and isocortex, hinting to the involvement in a wide range of sensory, emotional, and cognitive processing of gustatory stimuli.

## Food processing in the insular cortex

The insular cortex is integrated in a distinct network responsible for the neural control of appetite and the regulation of energy balance. Whereas the hypothalamus represents the major homeostatic player, the insular cortex is integrated in the neural system which is involved in the processing of external sensory information tightly linked to reward processing (Berthoud, 2011). Therefore, the insular cortex activity also contributes to the hedonic system.

**Figure 1:**
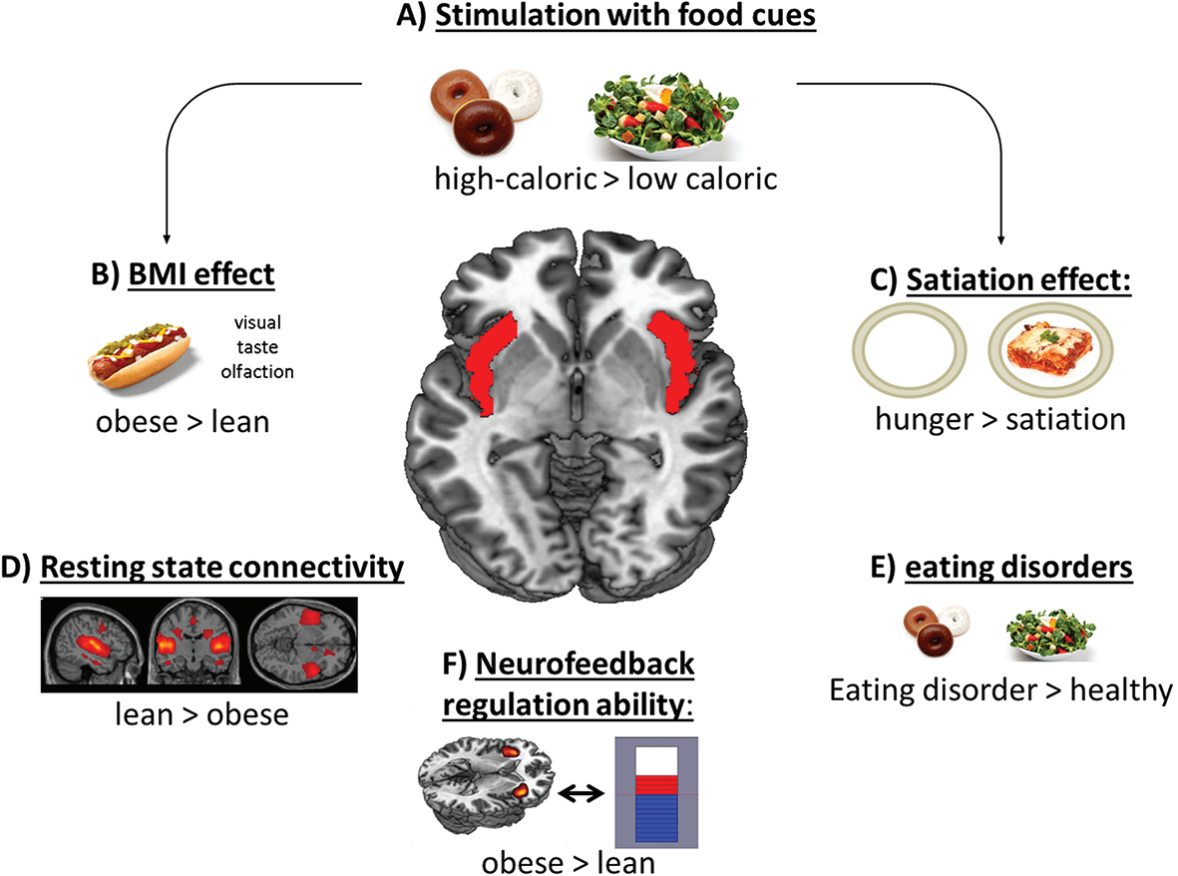
**Scheme of the contribution of the insular cortex in food-related processes.** Especially the anterior and mid-dorsal part of the insular cortex respond to **(A)** high-caloric food cues and show **(B)** increased activation in obese subjects and **(C)** in a hungry condition after stimulation with food items. **(D)** Lean subjects showed higher resting state connectivity pattern in the salience network, including the insular cortex. **(E)** Also patients suffering from an eating disorder show enhanced activation in this region. **(F)** Obese subjects’ regulation ability during an fMRI based neurofeedback paradigm is higher compared to lean subjects.

Several neuroimaging studies emphasized the functional contribution of the anterior insula in gustatory perception (Small et al., 2003; Veldhuizen et al., 2011; Figure 1A), which is represented in the processing of visually presented (Porubska et al., 2006; Frank et al., 2010), tasted or smelled food stimuli (De Araujo et al., 2003), and also in food craving (Pelchat, 1997; Pelchat et al., 2004). Eating per se is a multimodal experience, including taste, olfaction, smell, and somatosensory inputs (De Araujo and Simon, 2009). As part of the primary taste and primary olfactory cortex (Rolls, 2006; Small, 2010), the anterior insula is also highly responsive to different flavors (Rolls, 2005; Small, 2012; Small and Green, 2012). Sensory food-related inputs are combined in the anterior insula (Small, 2012), resulting in increased activation of this region after stimulation with a specific flavor (Small et al., 1999). Small and Prescott (2005) describe overlapping activation in the anterior insula after independent stimulation with taste and odor cues. Besides the taste component, transferred from the taste buds on the tongue to the primary taste cortex, the aroma of food is also experienced olfactorily via the retronasal route (Ruijschop et al., 2009; Small and Green, 2012).

Also, the texture and viscosity of ingested food is represented in the anterior and mid-insular cortex. Here, the activation changes according to the viscosity of a stimulus (De Araujo and Rolls, 2004; Alonso et al., 2007).

Besides components like taste, aroma and texture, also the amount of fat influences the activity in this gustatory and hedonic region. A recent fMRI study (Frank et al., 2012b), investigating the effect of a high- and low-fat meal on the cerebral blood flow, revealed a differential influence of fat on the mid-anterior insular cortex and the hypothalamus. The activity in the hypothalamus, representing the homeostatic system in the brain, decreased after intake of a high-fat meal, whereas the insular cortex activity increased after intake of the low-fat meal. This suggests an interaction of the homeostatic and the gustatory system, which might be mediated by the fat content of the meal.

The processing of food also includes the internal evaluation of the ingested, seen or smelled nutrients. The evaluative component includes interoceptive awareness, which is as well associated with insular processing (Craig, 2009). On a behavioral level, it was shown that good cardiac awareness, as a marker of interoception, is inversely related to the experienced fullness and myoelectric gastric activity after water load (Herbert et al., 2012). On the neuronal level, gastric distention without actual food intake leads to increased activation in the posterior insular cortex (Wang et al., 2008). Such findings corroborate the integrative function of the insular cortex.

A recent meta-analysis by Brooks et al. (2013) report decreased activation in obese compared to lean subjects in the mid-insular cortex, a region shown to be involved in interoceptive awareness (Simmons et al., 2012). The reduced awareness of the bodily state and, therefore, also for appetite signals of the gut and brain might be a reason for obese to consume more food in order to feel the interoceptive cues from the body in the same way normal-weight people do (Brooks et al., 2013).

Craig (2005) already proposed laterality differences in introceptive perception related to emotional processing. In a previous study a stronger impairment in taste functions in patients suffering from a lesion in the left anterior insular cortex compared to patients with a lesion in the right anterior insula (Stevenson et al., 2013), was shown. Furthermore, there is evidence that pleasant odors are rather processed in the left hemisphere and unpleasant odors in the right hemisphere (Henkin and Levy, 2001). However, further evidence is needed to understand possible hemispheric relationships of insular functions in more detail.

## Eating disorders

It has been shown that bulimia nervosa (BN) patients exhibit increased insula activation to high-caloric food pictures in comparison to overweight and normal weight control subjects (Schienle et al., 2009). This difference is possibly due to the enhanced autonomic arousal that appetizing food pictures elicit in BN. Increased insula activation was also shown in anorexia nervosa (AN) patients compared to healthy subjects when contrasting pictures of high- versus low-calorie drinks (Nunn et al., 2011; Figure 1E). In contrast, after the ingestion of chocolate milk in a hungry state, AN patients exhibited less activation in the insula than control subjects (Vocks et al., 2011). Of special importance is the change in insula function when women recovered from AN or BN. While AN recovered patients showed a decreased anterior insula activity after drinking sweet tastes (Wagner et al., 2008; Oberndorfer et al., 2013), BN recovered patients revealed an enhanced insula response in relation to weight matched controls (Oberndorfer et al., 2013). The different activation patterns may result from an altered processing of hunger or reward signals and a misinterpretation of internal feeling and feeding states that lead to exaggerated or restricted eating behavior even after treatment.

## Differential activation in lean and obese

Neuroimaging studies investigating food processing, by means of visual stimulation, have shown enhanced insula activity in obese compared to lean subjects (Figure 1B). Specifically, obese subjects were found to show higher left anterior and right mid-insular activity compared to lean control subjects in respone to food cues (Scharmuller et al., 2012). Also, Rothemund et al. (2007) and Stoeckel et al. (2008) reported enhanced activation in response to high-caloric food pictures in obese women in the anterior insula. In adolescent girls, the activation in the anterior insula correlated positively with the BMI during the orientation to food cues (Yokum et al., 2011). Studies investigating linear relationships of BMI with brain functions showed heightened activity in the anterior insula and the adjacent frontal operculum with increasing BMI (Ziauddeen et al., 2012).

Beside visual stimulation with food items, studies using oral food cues have also shown the insular cortex to be vital for food intake. While hunger resulted in a regional cerebral blood flow (rCBF) increase after administration of 2 mL of drinking water, satiation has been associated with a decrease in insular rCBF, suggesting that the reaction of the insular cortex to sensory experiences is affected by hunger (Del Parigi et al., 2002; Figure 1C). However, this decrease after satiation was more pronounced in obese compared to lean subjects (Gautier et al., 2000, 2001). Additionally, obese subjects revealed an enhanced sensory experience in the mid-dorsal insula to a liquid meal after a prolonged fast (Delparigi et al., 2005). Concomitantly, these results point to abnormal gustatory processing in obesity in response to a meal as well as to the sensory processing of food. The combination of a sweetened drink with the stimulation with food pictures also revealed enhanced anterior insula activation in obese subjects (Connolly et al., 2013), supporting the integration of multimodal stimuli in this area. Generally, the anterior insular cortex is highly responsive to food intake and anticipated food intake, a response that is more pronounced in obese (Stice et al., 2008, 2009).

Besides changes in activity, the insular cortex also revealed significant changes in functional connectivity in obese compared to lean subjects during resting-state and in response to food cues (Figure 1D). As such, the anterior insula has significant functional connections to several frontal, temporal, and parietal areas, in particular to the OFC, inferior frontal cortex and to the ACC in normal weight subjects (Taylor et al., 2009; Deen et al., 2011). In contrast, obese subjects revealed decreased functional connectivity in the insular cortex during resting-state (Kullmann et al., 2012), and increased functional and effective connectivity in response to food cues especially to striatal regions (Garcia-Garcia et al., 2012; Nummenmaa et al., 2012; Kullmann et al., 2013).

## The problem of weight loss maintenance

When facing the problem of obesity, one pressing question is how to effectively lose and maintain body weight. Successful weight loss maintainers show a greater bilateral insula response after orosensory stimulation with food cues (Sweet et al., 2012). Interestingly, the response to visually presented food items in the insular cortex seems to be predictive for the weight loss outcome. Less successful patients in a weight loss program showed higher insular activation pre- and post-treatment (Murdaugh et al., 2012). After successful weight-loss maintenance achieved by bariatric surgery, neuroimaging studies have shown that brain activations after food intake or visual stimulation with food cues are comparable with lean subjects (Van De Sande-Lee et al., 2011; Frank et al., 2013). Also in motivational and reward-related regions (including the insular cortex) stimulation with food pictures showed decreased activation after gastric banding (Bruce et al., 2012).

## Neurofeedback as a possible therapeutic approach

Regarding the increasing prevalence for obesity and the frequent failure of weight maintenance after weight loss, new therapeutic approaches are urgently needed. Therefore, it is intriguing to speculate about possible biofeedback strategies. Food-specific electrodermal biofeedback leads to increased food-related self-efficacy and reduced perceived stress (Teufel et al., 2013). Morewedge et al. (2010) reported that food consumption can be reduced by thoughts for food in lean subjects. The focus on food during eating enhances memory for a meal to later time points and reduce later food intake (Higgs and Donohoe, 2011). One innovative approach that might support the effort of obesity treatment is an fMRI-based neurofeedback training, which allows the voluntary regulation of specific brain regions (Birbaumer et al., 2013). Considering the multimodal functions of the insular cortex and its importance for food reward, the anterior insula seems to be an appropriate target for real-time fMRI (rtfMRI) neurofeedback. In a previous rtfMRI study, addressing the anterior insular cortex as the region of interest (ROI), lean participants learned to regulate this region voluntarily within one day over four training sessions (Caria et al., 2007). In a follow-up study, this group demonstrated that successful regulation compared to no regulation of the anterior insular cortex resulted in increased negative valence ratings of emotional pictures (Caria et al., 2010). Furthermore, it was shown that effective connectivity between the anterior insular cortex and areas involved in emotional processing were strongest in the best regulation session (Veit et al., 2012). In a recent study, we addressed insular neurofeedback training in obese subjects (Frank et al., 2012a). During the training sessions, all obese participants were able to regulate the activity, whereas four out of eleven participants of the lean group were not able to successfully regulate the anterior insula (Figure 1F). Investigating underlying neural connectivity processes, lean regulators in comparison to obese regulators showed stronger functional connectivity in cingular and temporal cortices during regulation. Therefore, lean and obese subjects seem to recruit differential neural networks to perform a voluntary regulation of primary gustatory systems.

## Conclusion

In conclusion, the insular cortex, especially the anterior part, is a multimodal and integrative area for the processing of food-related items. Central gustatory processes are tightly linked to interoception represented in reduced awareness of bodily signals including satiety signals. Therefore, interoception is associated with eating behavior and consequently also with obesity and eating disorders. In fact, multiple functions integrated in the insular cortex correlate and interact with gustatory processes. It has been shown, that obese subjects show higher responses in the anterior insular cortex to food cues independent of the modality (taste, visual). Moreover, rtfMRI guided neurofeedback training of the insular cortex raises the possibility to modify eating behavior.

## Conflict of interest statement

The authors declare that the research was conducted in the absence of any commercial or financial relationships that could be construed as a potential conflict of interest.

## References

[B1] Alonso B. D. C. Marciani L. Head K. Clark P. Spiller R. C. Rayment P. (2007). Functional magnetic resonance imaging assessment of the cortical representation of oral viscosity. J. Texture Stud. 38, 725–737 10.1111/j.1745-4603.2007.00122.x

[B2] Berthoud H. R. (2011). Metabolic and hedonic drives in the neural control of appetite: who is the boss? Curr. Opin. Neurobiol. 21, 888–896 10.1016/j.conb.2011.09.004 21981809 PMC3254791

[B3] Birbaumer N. Ruiz S. Sitaram R. (2013). Learned regulation of brain metabolism. Trends Cogn. Sci. 17, 295–302 10.1016/j.tics.2013.04.009 23664452

[B4] Brooks S. J. Cedernaes J. Schioth H. B. (2013). Increased prefrontal and parahippocampal activation with reduced dorsolateral prefrontal and insular cortex activation to food images in obesity: a meta-analysis of FMRI studies. PLoS One 8:e60393 10.1371/journal.pone.0060393 23593210 PMC3622693

[B5] Bruce J. M. Hancock L. Bruce A. Lepping R. J. Martin L. Lundgren J. D. (2012). Changes in brain activation to food pictures after adjustable gastric banding. Surg. Obes. Relat. Dis. 8, 602–608 10.1016/j.soard.2011.07.006 21996599

[B6] Caria A. Sitaram R. Veit R. Begliomini C. Birbaumer N. (2010). Volitional control of anterior insula activity modulates the response to aversive stimuli. A real-time functional magnetic resonance imaging study. Biol. Psychiatry 68, 425–432 10.1016/j.biopsych.2010.04.020 20570245

[B7] Caria A. Veit R. Sitaram R. Lotze M. Weiskopf N. Grodd W. (2007). Regulation of anterior insular cortex activity using real-time fMRI. Neuroimage 35, 1238–1246 10.1016/j.neuroimage.2007.01.018 17336094

[B8] Connolly L. Coveleskie K. Kilpatrick L. A. Labus J. S. Ebrat B. Stains J. (2013). Differences in brain responses between lean and obese women to a sweetened drink. Neurogastroenterol. Motil. 25, 579–e460 10.1111/nmo.12125 23566308 PMC4114731

[B9] Craig A. D. (2005). Forebrain emotional asymmetry: a neuroanatomical basis? Trends Cogn. Sci. 9, 566–571 10.1016/j.tics.2005.10.005 16275155

[B10] Craig A. D. (2009). How do you feel now? The anterior insula and human awareness. Nat. Rev. Neurosci. 10, 59–70 10.1038/nrn2555 19096369

[B11] De Araujo I. E. Rolls E. T. (2004). Representation in the human brain of food texture and oral fat. J. Neurosci. 24, 3086–3093 10.1523/jneurosci.0130-04.2004 15044548 PMC6729847

[B12] De Araujo I. E. Rolls E. T. Kringelbach M. L. Mcglone F. Phillips N. (2003). Taste-olfactory convergence, and the representation of the pleasantness of flavour, in the human brain. Eur. J. Neurosci. 18, 2059–2068 10.1046/j.1460-9568.2003.02915.x 14622239

[B13] De Araujo I. E. Simon S. A. (2009). The gustatory cortex and multisensory integration. Int. J. Obes. (Lond) 33 Suppl. 2, S34–S43 10.1038/ijo.2009.70 19528978 PMC2726647

[B14] Deen B. Pitskel N. B. Pelphrey K. A. (2011). Three systems of insular functional connectivity identified with cluster analysis. Cereb. Cortex 21, 1498–1506 10.1093/cercor/bhq186 21097516 PMC3116731

[B15] Del Parigi A. Gautier J. F. Chen K. Salbe A. D. Ravussin E. Reiman E. (2002). Neuroimaging and obesity: mapping the brain responses to hunger and satiation in humans using positron emission tomography. Ann. N Y Acad. Sci. 967, 389–397 10.1111/j.1749-6632.2002.tb04294.x 12079866

[B16] Delparigi A. Chen K. Salbe A. D. Reiman E. M. Tataranni P. A. (2005). Sensory experience of food and obesity: a positron emission tomography study of the brain regions affected by tasting a liquid meal after a prolonged fast. Neuroimage 24, 436–443 10.1016/j.neuroimage.2004.08.035 15627585

[B17] Frank S. Laharnar N. Kullmann S. Veit R. Canova C. Hegner Y. L. (2010). Processing of food pictures: influence of hunger, gender and calorie content. Brain Res. 1350, 159–166 10.1016/j.brainres.2010.04.030 20423700

[B18] Frank S. Lee S. Preissl H. Schultes B. Birbaumer N. Veit R. (2012a). The obese brain athlete: self-regulation of the anterior insula in adiposity. PLoS One 7:e42570 10.1371/journal.pone.0042570 22905151 PMC3414443

[B19] Frank S. Linder K. Kullmann S. Heni M. Ketterer C. Cavusoglu M. (2012b). Fat intake modulates cerebral blood flow in homeostatic and gustatory brain areas in humans. Am. J. Clin. Nutr. 95, 1342–1349 10.3945/ajcn.111.031492 22572644

[B20] Frank S. Wilms B. Veit R. Ernst B. Thurnheer M. Kullmann S. (2013). Altered brain activity in severely obese women may recover after Roux-en Y gastric bypass surgery. Int. J. Obes. (Lond) [Epub ahead of print]. 10.1038/ijo.2013.60 23711773

[B21] Gallay D. S. Gallay M. N. Jeanmonod D. Rouiller E. M. Morel A. (2012). The insula of reil revisited: multiarchitectonic organization in macaque monkeys. Cereb. Cortex 22, 175–190 10.1093/cercor/bhr104 21613468 PMC3236796

[B22] Garcia-Garcia I. Jurado M. A. Garolera M. Segura B. Sala-Llonch R. Marques-Iturria I. (2012). Alterations of the salience network in obesity: a resting-state fMRI study. Hum. Brain Mapp. [Epub ahead of print]. 10.1002/hbm.22104 22522963 PMC6870073

[B23] Gautier J. F. Chen K. Salbe A. D. Bandy D. Pratley R. E. Heiman M. (2000). Differential brain responses to satiation in obese and lean men. Diabetes 49, 838–846 10.2337/diabetes.49.5.838 10905495

[B24] Gautier J. F. Del Parigi A. Chen K. Salbe A. D. Bandy D. Pratley R. E. (2001). Effect of satiation on brain activity in obese and lean women. Obes. Res. 9, 676–684 10.1038/oby.2001.92 11707534

[B25] Henkin R. I. Levy L. M. (2001). Lateralization of brain activation to imagination and smell of odors using functional magnetic resonance imaging (fMRI): left hemispheric localization of pleasant and right hemispheric localization of unpleasant odors. J. Comput. Assist. Tomogr. 25, 493–514 10.1097/00004728-200107000-00001 11473178

[B26] Herbert B. M. Muth E. R. Pollatos O. Herbert C. (2012). Interoception across modalities: on the relationship between cardiac awareness and the sensitivity for gastric functions. PLoS One 7:e36646 10.1371/journal.pone.0036646 22606278 PMC3350494

[B27] Higgs S. Donohoe J. E. (2011). Focusing on food during lunch enhances lunch memory and decreases later snack intake. Appetite 57, 202–206 10.1016/j.appet.2011.04.016 21569808

[B28] Kullmann S. Heni M. Veit R. Ketterer C. Schick F. Häring H. U. (2012). The obese brain: association of body mass index and insulin sensitivity with resting state network functional connectivity. Hum. Brain Mapp. 33, 1052–1061 10.1002/hbm.21268 21520345 PMC6870244

[B29] Kullmann S. Pape A. A. Heni M. Ketterer C. Schick F. Haring H. U. (2013). Functional network connectivity underlying food processing: disturbed salience and visual processing in overweight and obese adults. Cereb. Cortex 23, 1247–1256 10.1093/cercor/bhs124 22586138

[B30] Kurth F. Zilles K. Fox P. T. Laird A. R. Eickhoff S. B. (2010). A link between the systems: functional differentiation and integration within the human insula revealed by meta-analysis. Brain Struct. Funct. 214, 519–534 10.1007/s00429-010-0255-z 20512376 PMC4801482

[B31] Menon V. Uddin L. Q. (2010). Saliency, switching, attention and control: a network model of insula function. Brain Struct. Funct. 214, 655–667 10.1007/s00429-010-0262-0 20512370 PMC2899886

[B32] Mesulam M. M. Mufson E. J. (1985). “The insula of reil in man and monkey: architectonics, connectivity and function,” in Cerebral Cortex, eds Peters A. Jones E. O. (New York: Plenum Press), 179–226

[B33] Morewedge C. K. Huh Y. E. Vosgerau J. (2010). Thought for food: imagined consumption reduces actual consumption. Science 330, 1530–1533 10.1126/science.1195701 21148388

[B34] Murdaugh D. L. Cox J. E. Cook E. W., 3rd Weller R. E. (2012). fMRI reactivity to high-calorie food pictures predicts short- and long-term outcome in a weight-loss program. Neuroimage 59, 2709–2721 10.1016/j.neuroimage.2011.10.071 22332246 PMC3287079

[B35] Nummenmaa L. Hirvonen J. Hannukainen J. C. Immonen H. Lindroos M. M. Salminen P. (2012). Dorsal striatum and its limbic connectivity mediate abnormal anticipatory reward processing in obesity. PLoS One 7:e31089 10.1371/journal.pone.0031089 22319604 PMC3272045

[B36] Nunn K. Frampton I. Fuglset T. S. Torzsok-Sonnevend M. Lask B. (2011). Anorexia nervosa and the insula. Med. Hypotheses 76, 353–357 10.1016/j.mehy.2010.10.038 21087828

[B37] Oberndorfer T. A. Frank G. K. Simmons A. N. Wagner A. Mccurdy D. Fudge J. L. (2013). Altered insula response to sweet taste processing after recovery from anorexia and bulimia nervosa. Am. J. Psychiatry [Epub ahead of print]. 10.1176/appi.ajp.2013.11111745 23732817 PMC3971875

[B38] Pelchat M. L. (1997). Food cravings in young and elderly adults. Appetite 28, 103–113 10.1006/appe.1996.0063 9158846

[B39] Pelchat M. L. Johnson A. Chan R. Valdez J. Ragland J. D. (2004). Images of desire: food-craving activation during fMRI. Neuroimage 23, 1486–1493 10.1016/j.neuroimage.2004.08.023 15589112

[B40] Porubska K. Veit R. Preissl H. Fritsche A. Birbaumer N. (2006). Subjective feeling of appetite modulates brain activity: an fMRI study. Neuroimage 32, 1273–1280 10.1016/j.neuroimage.2006.04.216 16815041

[B41] Rolls E. T. (2005). Taste, olfactory, and food texture processing in the brain, and the control of food intake. Physiol. Behav. 85, 45–56 10.1016/j.physbeh.2005.04.012 15924905

[B42] Rolls E. T. (2006). Brain mechanisms underlying flavour and appetite. Philos. Trans. R. Soc. Lond. B Biol. Sci. 361, 1123–1136 10.1098/rstb.2006.1852 16815796 PMC1642694

[B43] Rothemund Y. Preuschhof C. Bohner G. Bauknecht H. C. Klingebiel R. Flor H. (2007). Differential activation of the dorsal striatum by high-calorie visual food stimuli in obese individuals. Neuroimage 37, 410–421 10.1016/j.neuroimage.2007.05.008 17566768

[B44] Ruijschop R. M. Boelrijk A. E. De Graaf C. Westerterp-Plantenga M. S. (2009). Retronasal aroma release and satiation: a review. J. Agric. Food Chem. 57, 9888–9894 10.1021/jf901445z 19817424

[B45] Scharmuller W. Ubel S. Ebner F. Schienle A. (2012). Appetite regulation during food cue exposure: a comparison of normal-weight and obese women. Neurosci. Lett. 518, 106–110 10.1016/j.neulet.2012.04.063 22580204

[B46] Schienle A. Schafer A. Hermann A. Vaitl D. (2009). Binge-eating disorder: reward sensitivity and brain activation to images of food. Biol. Psychiatry 65, 654–661 10.1016/j.biopsych.2008.09.028 18996508

[B47] Simmons W. K. Avery J. A. Barcalow J. C. Bodurka J. Drevets W. C. Bellgowan P. (2012). Keeping the body in mind: insula functional organization and functional connectivity integrate interoceptive, exteroceptive, and emotional awareness. Hum. Brain Mapp. [Epub ahead of print]. 10.1002/hbm.22113 22696421 PMC6870113

[B48] Small D. M. (2010). Taste representation in the human insula. Brain Struct. Funct. 214, 551–561 10.1007/s00429-010-0266-9 20512366

[B49] Small D. M. (2012). Flavor is in the brain. Physiol. Behav. 107, 540–552 10.1016/j.physbeh.2012.04.011 22542991

[B50] Small D. M. Green B. G. (2012). “A proposed model of a flavor modality,” in The Neural Bases of Multisensory Processes, eds Murray M. M. Wallace M. T. (Boca Raton (FL): CRC Press), 717–738

[B51] Small D. M. Gregory M. D. Mak Y. E. Gitelman D. Mesulam M. M. Parrish T. (2003). Dissociation of neural representation of intensity and affective valuation in human gustation. Neuron 39, 701–711 10.1016/s0896-6273(03)00467-7 12925283

[B52] Small D. M. Prescott J. (2005). Odor/taste integration and the perception of flavor. Exp. Brain Res. 166, 345–357 10.1007/s00221-005-2376-9 16028032

[B53] Small D. M. Zald D. H. Jones-Gotman M. Zatorre R. J. Pardo J. V. Frey S. (1999). Human cortical gustatory areas: a review of functional neuroimaging data. Neuroreport 10, 7–14 10.1097/00001756-199901180-00002 10094124

[B54] Stevenson R. J. Miller L. A. Mcgrillen K. (2013). The lateralization of gustatory function and the flow of information from tongue to cortex. Neuropsychologia 51, 1408–1416 10.1016/j.neuropsychologia.2013.04.010 23628369

[B55] Stice E. Spoor S. Bohon C. Veldhuizen M. G. Small D. M. (2008). Relation of reward from food intake and anticipated food intake to obesity: a functional magnetic resonance imaging study. J. Abnorm. Psychol. 117, 924–935 10.1037/a0013600 19025237 PMC2681092

[B56] Stice E. Spoor S. Ng J. Zald D. H. (2009). Relation of obesity to consummatory and anticipatory food reward. Physiol. Behav. 97, 551–560 10.1016/j.physbeh.2009.03.020 19328819 PMC2734415

[B57] Stoeckel L. E. Weller R. E. Cook E. W., 3rd Twieg D. B. Knowlton R. C. Cox J. E. (2008). Widespread reward-system activation in obese women in response to pictures of high-calorie foods. Neuroimage 41, 636–647 10.1016/j.neuroimage.2008.02.031 18413289

[B58] Sweet L. H. Hassenstab J. J. Mccaffery J. M. Raynor H. A. Bond D. S. Demos K. E. (2012). Brain response to food stimulation in obese, normal weight, and successful weight loss maintainers. Obesity (Silver Spring) 20, 2220–2225 10.1038/oby.2012.125 22569002 PMC3483466

[B59] Taylor K. S. Seminowicz D. A. Davis K. D. (2009). Two systems of resting state connectivity between the insula and cingulate cortex. Hum. Brain Mapp. 30, 2731–2745 10.1002/hbm.20705 19072897 PMC6871122

[B60] Teufel M. Stephan K. Kowalski A. Kasberger S. Enck P. Zipfel S. (2013). Impact of biofeedback on self-efficacy and stress reduction in obesity: a randomized controlled pilot study. Appl. Psychophysiol. Biofeedback [Epub ahead of print]. 10.1007/s10484-013-9223-8 23760668

[B61] Van De Sande-Lee S. Pereira F. R. Cintra D. E. Fernandes P. T. Cardoso A. R. Garlipp C. R. (2011). Partial reversibility of hypothalamic dysfunction and changes in brain activity after body mass reduction in obese subjects. Diabetes 60, 1699–1704 10.2337/db10-1614 21515852 PMC3114393

[B62] Veit R. Singh V. Sitaram R. Caria A. Rauss K. Birbaumer N. (2012). Using real-time fMRI to learn voluntary regulation of the anterior insula in the presence of threat-related stimuli. Soc. Cogn. Affect. Neurosci. 7, 623–634 10.1093/scan/nsr061 21983794 PMC3427870

[B63] Veldhuizen M. G. Albrecht J. Zelano C. Boesveldt S. Breslin P. Lundstrom J. N. (2011). Identification of human gustatory cortex by activation likelihood estimation. Hum. Brain Mapp. 32, 2256–2266 10.1002/hbm.21188 21305668 PMC3123671

[B64] Vocks S. Herpertz S. Rosenberger C. Senf W. Gizewski E. R. (2011). Effects of gustatory stimulation on brain activity during hunger and satiety in females with restricting-type anorexia nervosa: an fMRI study. J. Psychiatr. Res. 45, 395–403 10.1016/j.jpsychires.2010.07.012 20709330

[B65] Wager T. D. Barrett L. F. (2004). From affect to control: functional specialization of the insula in motivation and regulation. *Published online at PsycExtra.*

[B66] Wagner A. Aizenstein H. Mazurkewicz L. Fudge J. Frank G. K. Putnam K. (2008). Altered insula response to taste stimuli in individuals recovered from restricting-type anorexia nervosa. Neuropsychopharmacology 33, 513–523 10.1038/sj.npp.1301443 17487228

[B67] Wang G. J. Tomasi D. Backus W. Wang R. Telang F. Geliebter A. (2008). Gastric distention activates satiety circuitry in the human brain. Neuroimage 39, 1824–1831 10.1016/j.neuroimage.2007.11.008 18155924

[B68] Yokum S. Ng J. Stice E. (2011). Attentional bias to food images associated with elevated weight and future weight gain: an FMRI study. Obesity 19, 1775–1783 10.1038/oby.2011.168 21681221 PMC4007087

[B69] Ziauddeen H. Farooqi I. S. Fletcher P. C. (2012). Obesity and the brain: how convincing is the addiction model? Nat. Rev. Neurosci. 13, 279–286 10.1038/nrn3212 22414944

